# Clinical significance of checkpoint regulator “Programmed death ligand-1 (PD-L1)” expression in meningioma: review of the current status

**DOI:** 10.1007/s11060-020-03584-8

**Published:** 2021-02-21

**Authors:** Shirin Karimi, Sheila Mansouri, Farshad Nassiri, Severa Bunda, Olivia Singh, Priscilla K. Brastianos, Ian F. Dunn, Gelareh Zadeh

**Affiliations:** 1 MacFeeters-Hamilton Center for Neuro-Oncology Research, Princess Margaret Cancer Center, 14-701, Toronto Medical Discovery Tower (TMDT), 101 College St, Toronto, ON M5G 1L7 Canada; 2 Division of Neurosurgery, University Health Network, University of Toronto, Toronto, ON Canada; 3 Massachusetts General Hospital, Harvard Medical School, Boston, MA USA; 4 Department of Neurosurgery, Brigham and Women’s Hospital, Boston, MA USA

**Keywords:** Meningioma, PD-L1, Immunohistochemistry, Checkpoint

## Abstract

**Introduction:**

Meningioma is the most common primary brain tumor. Most meningiomas are benign; however, a subset of these tumors can be aggressive, presenting with early or multiple tumor recurrences that are refractory to neurosurgical resection and radiotherapy. There is no standard systemic therapy for these patients, and post-surgical management of these patients is usually complicated due to lack of accurate prediction for tumor progression.

**Methods:**

In this review, we summarise the crucial immunosuppressive role of checkpoint regulators, including PD-1 and PD-L1 interacting in the tumor microenvironment, which has led to efforts aimed at targeting this axis.

**Results:**

Since their discovery, checkpoint inhibitors have significantly improved the outcome in many types of cancers. Currently, targeted therapy for PD-1 and PD-L1 proteins are being tested in several ongoing clinical trials for brain tumors such as glioblastoma. More recently, there have been some reports implicating increased PD-L1 expression in high-grade (WHO grades II and III) meningiomas. Several clinical trials are underway to assess the efficacy of checkpoint inhibitors in the therapeutic management of patients with aggressive meningiomas. Here, we review the immune suppressive microenvironment in meningiomas, and then focus on clinical and pathological characterization and tumor heterogeneity with respect to PD-L1 expression as well as challenges associated with the assessment of PD-L1 expression in meningioma.

**Conclusion:**

We conclude with a brief review of ongoing clinical trials using checkpoint inhibitors for the treatment of high-grade and refractory meningiomas.

## Background

Meningiomas arise from the arachidonic cap cells of the meninges and comprise the most common primary brain tumors in adults. The most recent WHO classification is based entirely on histologic criteria and stratifies tumors into benign (WHO grade I), atypical (WHO grade II), and anaplastic (WHO grade III) tumors [1]. Although most histologically benign meningiomas can be cured by neurosurgical resection, patients with high-grade tumors often experience rapid tumor progression and early recurrence despite surgical resection and radiotherapy. The estimated progression-free survival (PFS) at 10 years for meningioma WHO grades I, II, and III are 75–90%, 23–78%, and 0%, respectively [2]. Therefore, tumor recurrence presents a major clinical challenge for the management of these patients, even in WHO grade I meningioma [2, 3]. At present, there are no approved systemic therapies for patients with refractory meningiomas [3, 4]. A better understanding of the biology of tumor progression may lead to the identification of effective systemic treatments and improve outcomes for meningioma patients with refractory tumors. Recent investigations have identified biologically significant somatic mutations, chromosomal copy number aberrations, and epigenetic alterations that drive meningioma growth or progression.

The main genetic alteration in meningioma is the deletion of genes on chromosome 22q, in particular, loss-of-function mutations in the neurofibromin 2/merlin/schwannomin (*NF2*) gene, dividing meningiomas into *NF2*-mutant (60%) and wild-type groups [2, 5]. Importantly, the majority of meningiomas with *NF2* mutations demonstrate chromosomal instability and increased risk of tumor progression into high-grade meningiomas [6]. Most of the canonical non-*NF2* mutations (e.g., *TRAF7*, *KLF4*, *AKT1*, and *SMO*) are mutually exclusive of *NF2* mutations [7]. Based on these molecular features, several clinical trials in NF-2 wild type meningiomas, including targeted therapy for actionable mutations in *PIK3CA*, *SMO*, and *AKT1* are ongoing (NCT02523014) [3, 4]. However, only a subset of meningiomas harbor clinically actionable alterations, and many non-*NF2* mutations in high-grade tumors are not targetable; these tumors do, however, demonstrate complex copy number alterations including several chromosomal losses and gains as well as higher mutational burden and neoantigen load [8]. In addition to genomic alterations, it has been recently demonstrated that DNA methylation status can highly be predictive of tumor recurrence, providing an avenue for management of the highest-risk patients [9, 10].

Herein, we will review the immuno-oncology landscape of checkpoint PD-L1 expression in meningioma and summarize the clinical trials using checkpoint inhibitors to treat the selected meningiomas with an aggressive clinical course.

## Meningioma tumor immune microenvironment

The interaction between tumor and host immune response is complex and has been the primary focus in immune-oncology research. The tumor immune microenvironment has two essential components: immune cells and regulatory checkpoints. The body’s immune system identifies cancers as “foreign,” mounting an immune response primarily through T helper and cytotoxic T cells. Tumor antigens presented by the major histocompatibility complexes by antigen-presenting cells (APCs) result in T cell activation. However, tumor cells evade the immune cell activity by inducing an immunosuppressive microenvironment, along with loss of antigenicity and immunogenicity [11].

Meningiomas, unlike intra-axial brain tumors, have a unique dural-based anatomical location with ready access to circulating immune cells due to the absence of the blood–brain barrier. Current studies in the immuno-oncology of meningiomas have shown that the tumor immune microenvironment might play a role in tumor progression. In particular, tumor macrophages are the predominant immune cells in meningiomas, followed by T (helper, cytotoxic, and Treg) cells, a small population of natural killer (NK) and B cells [12]. Tumor-associated macrophages with M2 phenotype contribute to tumor progression [13]. Interestingly, increased expression of CD163 on either M2 macrophages or tumor cells has been observed in meningiomas with atypical features correlating with worse clinical outcome [14].

Overall, the available literature suggests that WHO grades II–III meningiomas exemplify a relatively immunosuppressed status. The majority of low-grade meningioma show increased numbers of perivascular antigen-experienced T cells [15]. Recent work on tumor immune microenvironment demonstrated that WHO grading in meningioma negatively correlates with the proportion of CD4^+^, CD8^+^, and PD-1^+^ lymphocytes, along with increased numbers of Treg (FOXP3^+^) cells in the tumor [16]. Treg lymphocytes decrease the proliferation of T cells and modulate the host immunity against tumor cells [17]. B cells have occasionally been detected in meningiomas [23], while an increased number of mast cells has been detected, particularly in meningiomas with peritumoral edema [18].

In addition to the WHO grade, genetic and cytogenetic alterations may influence the tumor immune profile. In particular, it has been shown that meningiomas with isolated monosomy of chromosome 22 are associated with an increased proportion of tissue macrophages and NK cells compared to meningiomas with a diploid and complex karyotype [17]. Similarly, Adams et al. reported a relative decrease in the numbers of M2 macrophages in *AKT1 E17K* non-NF2 mutated meningiomas in comparison to *NF2* mutated meningioma, indicative of an immunosuppressive tumor microenvironment in the latter group [20]. These findings support the impact of somatic genetic alterations on tumor immune cell infiltration in the tumor microenvironment in meningioma.

## PD-L1 checkpoint expression in meningioma

The interaction between tumor and immune cells is under heavy regulation by immune checkpoints. The most important checkpoints include PD-1 expression on immune cells, and its primary ligand: programmed death-ligand 1 (PD-L1). *PDCDL1* gene on chromosome 9p encodes PD-L1 (CD274), which is a complex transmembrane protein [21, 22]. Other important checkpoints are cytotoxic T lymphocyte antigen 4 (CTLA-4) and its respective ligands CD80 (B7.1) and CD86 (B7.2) on immune cells [23]. Immune checkpoint inhibitors primarily release the immune system’s brakes to activate an anti-tumor immune response that may involve activated T cells targeting tumor cells [23, 24].

The development of immunotherapy with checkpoint inhibitors has dramatically improved patient outcomes for various cancer types. It has been shown that PD-L1 protein expression using immunohistochemistry is a reliable predictor for response to checkpoint inhibitor therapy and patients’ outcome in many types of cancers such as non-small cell lung carcinoma, melanoma and even in benign tumors including paraganglioma and pheochromocytoma [24–29]. However, the optimization and standardization of the IHC protocol, together with the analysis of this biomarker exert challenges in immunopathology. The antibody clone, the optimized protocol, scoring method, interobserver variability, different cut-offs, intensity, and percentage of membranous reactivity on tumor and immune cells can dramatically affect the histopathological assessment of PD-L1 and its predictive significance [30, 31].

Emerging data suggest that similar to peripheral nerve sheath tumors [32] and other primary brain tumors such as glioblastoma [33], PD-L1-induced immune-suppression may exert a biological role in the progression of meningioma to higher malignancy grade and possibly acts as a predictor of patient outcome. Several studies have compared PD-L1 expression across the three WHO grades of meningioma (Table 1). Du et al. characterized both tumor and immune cells in meningioma, and they demonstrated significantly greater PD-L1 protein expression on tumor cells in high WHO grades of meningioma, benign (40%), atypical (60%) and anaplastic (77–88%) [16]. Their analysis of PD-L1 mRNA expression confirmed the positive association between PD-L1 expression and grade. Furthermore, their results regarding decreased immune cell infiltration in the malignant meningioma supported the immune-suppressive tumor microenvironment in these tumors [16]. However, they did not find any association between PD-L1 expression on tumor cells and clinical outcome in their cohort.

**Table 1:** Summary of studies on PD-L1 IHC expression across three WHO grades of meningioma

Study (references)	Cohort (n)	PD-L1 Ab	PD-L1 (%)			Association with radiotherapy	Prognostic significance
			G I	G II	G III		
Du et al. [16]	173	Sinobiological	40	60	78–89	No	Not prognostic for RFS
Hans et al. [34]	96	Cell signaling	1	3	9	Yes, positive	Independent prognostic for OS
Karimi et al. [46]	93	Spring bioscience	27	47	67	No	Independent prognostic for RFS

*RFS* recurrence free survival, *OS* overall survival

Consistent with this study, Han et al. specifically analyzed the PD-L1 expression in both tumor and immune cells in meningioma using immune cell markers, including one of the macrophage biomarkers, CD68. The results confirmed that an increased percentage of CD68^−^/PD-L1^+^ tumor cells correlated with WHO grade using a tumor microarray of 96 meningiomas [34]. The authors did not detect any biologic significance for the relative proportion of CD68^+^/PD-L1^+^ cells in their cohort. This study demonstrated an independent prognostic significance for PD-L1 expression on tumor cells for overall survival in meningioma patients. Additionally, the authors found that PD-L1 expression increased from 2.5 to 6.8% following radiotherapy. Recently, Sato et al. reviewed the impact of radiotherapy on PD-L1 expression in cancer patients [35, 36]. It has been shown that activation of the JAK/STAT pathway induced by radiotherapy leads to the upregulation of PD-L1 expression in cancer cells [35]. A comprehensive understanding of the impact of radiotherapy on PD-L1 expression in meningioma is needed for developing combined immuno-radiotherapy in these patients.

More recently, our group assessed PD-L1 expression via immunohistochemistry, followed by quantification using both digital and visual scoring methods in whole tumor sections of 93 meningioma cases enriched with high-grade tumors (Karimi et al., *Scientific Reports*, *submitted*, *under review*). We did not specifically characterize the tumor and immune cells and instead, analyzed PD-L1 expression in the whole tumor. Prominent inter- and intra-tumoral heterogeneity with statistically significant PD-L1 protein expression was confirmed in high-grade meningiomas. PD-L1 protein expression displayed a patchy pattern, along with peri-vascular and peri-necrotic, membranous, and cytoplasmic immunoreactivity in both tumor and immune cells. PD-L1 positivity was confined to a small subpopulation of cells (median < 1% of cells, range 0–20% of cells), which is similar to the PD-L1 positivity reported in glioblastoma [33]. Furthermore, we demonstrated the prognostic significance of higher PD-L1 protein expression for worse recurrence-free survival (RFS) independent of WHO grade, the extent of resection, and maximum tumor diameter (Karimi et al., *Scientific Reports*, *submitted*, *under review*). Further, we did not find any association between the history of radiotherapy and PD-L1 expression in our cohort*.*

Another study reported on both systemic and local immunosuppression in WHO grade III meningiomas*.* Flow cytometric analysis of peripheral blood and IHC staining of tumoral tissue of the 53 meningiomas revealed significantly increased number of PD-L1-positive peripheral blood monocytes and intratumoral PD-L1 positive immune reactivity in WHO grades III cases. However, there was no prognostic significance for intra-tumoral PD-L1 expression to predict RFS.

In addition to WHO grade, the genetic background may also influence the extent of immune suppression in meningiomas. Hao et al. investigated the expression of immune checkpoint proteins in 92 skull-base meningiomas and found increased PD-L1 immunoreactivity in *TRAF7*-mutated tumors relative to wild-type [37]. Furthermore, elevated levels of two other checkpoint proteins, PD-L2 and B7-H3, have been reported in meningiomas harboring mutations in genes within the *PI3K*/*AKT*/*mTOR* pathway. However, these cases did not display significant changes in PD-L1 expression [38]. Additionally, these studies detected an increased number of CTLA4^+^/CD3^+^ lymphocytes in WHO grades II and III tumors with mutations in *PIK3CA* or *SMO*. Immunohistochemical analysis of PD-L1 expression in several tumor types associated with *NF-1* and *NF-2* gene mutations, including meningioma, schwannoma, and neurofibroma showed a variable degree of positivity across these tumors; specifically, they detected PD-L1 expression in 40% of NF2-mutated meningiomas [32]. These findings support the hypothesis that somatic genetic alterations in meningioma can potentially affect checkpoint protein expression and can be accounted for in patient stratification in future immunotherapy.

## Challenges associated with the assessment of PD-L1 expression by immunohistochemistry staining

Review of the key studies on PD-L1 expression in meningioma indicated a significant difference in the percentage of IHC positivity across three WHO grades and conflicting results in survival analyses or response to radiotherapy (Table 1). One of the main reasons for variations in the results may be associated with difficulties in the interpretation of PD-L1 positivity by IHC staining, as demonstrated in various malignancies [31]. Specifically, these differences may result from the quality of the antibody utilized, inter-observer variability, different cut-offs implemented, as well as different scoring methods applied (digital versus visual) [30, 39]. Furthermore, different immune cell populations could be an additional confounding factor in the assessment of PD-L1 positivity in meningiomas. For example, macrophages may comprise 50% of the PD-L1-expressing cells [30] and morphologically it is difficult to accurately differentiate PD-L1 positivity on tumor cells from macrophages. These factors could lead to conflicting results in these studies. Therefore, standardization of PD-L1 immunostaining protocol in meningioma will be helpful for translational research studies in the field of immuno-oncology.

## Novel immunotherapeutic approach for refractory meningiomas

While several clinical trials for single and combined immunotherapy are actively ongoing in glioblastoma [40], the therapeutic role of checkpoint inhibitors in meningiomas remains relatively under investigated.

The first case report showing the therapeutic effects of anti-PD-1 in meningioma was published in 2017. The patient was diagnosed with lung carcinoma and intracranial meningioma [41] and was treated with nivolumab (anti-PD-1 antibody), which is clinically used in the management of lung cancer patients. This treatment resulted in decreased meningioma tumor size, shedding light on the potential of immune checkpoint inhibitors for therapeutic management of aggressive meningioma. To the best of our knowledge, there are five ongoing clinical trials investigating checkpoint inhibitors in aggressive meningioma that measure the immune status outcome and PFS (Table 2) [4, 42]. Among these, the treatment plan of the three clinical trials is a combination of checkpoint inhibitors with radiotherapy (Table 2).

**Table 2:** The clinical trials investigating check point inhibitor in meningiomas

Identifier	Drug (check point targeted)	Combined therapy	Tumor	Outcome	Immune outcome measure
NCT03016091	Pembrolizomab (PD-1)		Refractory atypical and anaplastic meningioma, hemangiopericytoma	PFS	PD-L1 and PD-1 IHC expression
NCT03279692	Pembrolizomab (PD-1)		Recurrent or residual high-grade meningioma	PFS	
NCT02648997	Nivolumab (PD-1)/ipilimumab (CTLA4)	External beam RT (IMRT, 3D-CRT, or proton-beam radiation therapy)	Recurrent high-grade meningioma	PFS	Circulating immune cell subsets and cytokines, archival tumor, PD-L1 and PD-1 expressing tumor infiltrating lymphocytes, and archival tumor expression of immune gene expression signature
NCT03604978	Nivolumab (PD-1), ipilimumab (CTLA4)	Stereotactic radiosurgery	Recurrent, WHO grade II and III meningiomas	PFS	Molecular profiling assays such as tumor mutation burden and neoantigen load, immunophenotype changes of peripheral T-cells
NCT03267836	Avelumab (PD-L1)	Hypo fractionated proton radiation therapy	Recurrent, radiation refractory meningioma	PFS	Immunogenicity as measured by changes of CD8^+^/CD4^+^ tumor infiltrating lymphocytes (TILs)

The preliminary case report from the phase II study (NCT 02648997) using nivolumab (anti-PD-1 antibody) is promising. Dunn et al. observed a significant therapeutic benefit of nivolumab in a recurrent, atypical meningioma patient in this clinical trial [43]. Interestingly, the tumor mutation burden was high, and the PD-L1-positicity was restricted to the immune cells in the pre-treatment sample of the index tumor. To verify their observation in this case report, the authors reported a positive association between higher tumor mutation burden with anaplastic morphology, along with a higher proliferation index, and significant chromosomal abnormalities in high-grade meningioma cohort [8].

The four out of five clinical trials investigating immune checkpoint inhibitor use anti-PD-1 to treat aggressive meningiomas (Table 2). There is only one ongoing clinical trial which assesses the efficacy of anti-PD-L1 checkpoint inhibitor (NCT032678360). They combine post-operative avelumab (anti-PD-L1 antibody) and hypo-fractionated proton radiation therapy for recurrent, refractory meningiomas (NCT03267836). One of the advantages of using anti-PD-L1 in immunotherapy of meningioma is its secondary effect on the activation of NK cells. The anti-PD-L1 monoclonal antibody can exert dual adverse effects on tumor growth by direct inhibition of the PD-1/PD-L1 pathway, along with activation of NK cells [44].

Currently, most of the above-mentioned checkpoint inhibitor clinical trials in meningiomas investigate the predictive value of different immune biomarkers such as PD-L1 and PD-1 immunohistochemical staining in the tumor and immunophenotypes of tissue infiltrating lymphocytes. Although PD-L1 is one of the most important predictive biomarkers in several cancers, several studies in lung cancer have questioned its predictive significance for immunotherapy [27]. The results of the clinical trials investigating checkpoint inhibitors will provide the therapeutic effect of this type of immunotherapy on tumor progression in aggressive meningioma. These findings will also help us to determine the predictive biomarkers for response to checkpoint therapy in these patients.

## Conclusion

Refractory meningiomas are associated with high rates of morbidity and mortality. There is strong evidence that expression of the checkpoint regulator PD-L1 is associated with immunosuppression and correlates with WHO grade and possibly tumor recurrence in meningioma. The pattern of PD-L1 IHC expression is patchy with inter and intratumoral heterogeneity. Standardization and better pathologic characterization of PD-L1 IHC expression in meningioma will be helpful for translational and clinical research studies using this biomarker. There is some evidence of the impact of radiotherapy and somatic genetic alterations on the expression of PD-L1 in meningioma. Our current understanding suggests an immunosuppressive tumor microenvironment in high-grade meningiomas due to a decrease of immune cell component, high PD-L1 expression, and possibly high tumor mutation burden in these tumors. These findings provided a reasonable rationale for several ongoing checkpoint inhibitor therapy trials in high-grade and refractory meningiomas. The main predictive biomarkers that stratify patients for immunotherapy, including PD-L1 and PD-1 expression, tumor mutation burden, tumor-infiltrating lymphocytes, and immune gene signature, are under investigations in meningiomas [45].

The ongoing clinical trials using checkpoint inhibitors in meningioma will shed light on the therapeutic role of checkpoint inhibitors to control tumor progression and the significance of predictive biomarkers in immunotherapy of these patients. These data will aid us in developing a new immunotherapy approach for refractory and progressive meningiomas.
